# Persistence of H7N9 virus antibody response 2 years after infection

**DOI:** 10.1111/irv.12702

**Published:** 2019-12-19

**Authors:** Lin Yao, Guo‐Lin Wang, Li‐Ling Chen, Cheng Liu, Li‐Jun Duan, Gregory C. Gray, Mai‐Juan Ma

**Affiliations:** ^1^ State Key Laboratory of Pathogen and Biosecurity Beijing Institute of Microbiology and Epidemiology Beijing China; ^2^ Suzhou Municipal Center for Disease Control and Prevention Suzhou China; ^3^ Division of Infectious Diseases School of Medicine Global Health Institute Duke University Durham North Carolina; ^4^ Global Health Research Center Duke‐Kunshan University Kunshan China; ^5^ Program in Emerging Infectious Diseases Duke‐NUS Medical School Singapore City Singapore

**Keywords:** antibody response, H7N9 viruses, influenza A virus, persistence, serological

## Abstract

We measured antibodies against H7N9 virus 2 years after infection in 14 patients who were infected during October 2016‐September 2017. Approximately 2 years after infection, antibody titers ≥10 were detectable in 13 (92.9%) patients. Three (21.4%) of 14 patients had hemagglutination inhibition titers ≥40, and their geometric mean titer (GMT) was 20 (95% CI 15.7‐28.1), whereas 10 (71.4%) and all 14 (100%) of the 14 patients had titers ≥40, and GMTs at 34.4 (95% CI 25.7‐51.2) and 73.45 (54.7‐106.7) for neuraminidase inhibition and microneutralization antibodies, respectively. Our findings suggest that H7N9 infection may induce long‐term antibody response at least 2 years after infection.

## 1 INTRODUCTION

From October 1, 2016, through September 30, 2017 (5th wave), China experienced the largest outbreak of human avian influenza A(H7N9) virus infection since the virus was first detected in March 2013. Highly pathogenic strains of H7N9 viruses emerged and infected both humans and poultry.^1^ To control the transmission of H7N9 viruses from poultry to humans, beginning in September 2017 a mass poultry vaccination program was implemented. This campaign was highly effective in preventing H7N9 virus infection in both poultry and humans.^2^ However, novel, highly pathogenic subtypes (H7N9 and H7N2) adapted to ducks now pose new challenges to public health.^2^, ^3^, ^4^

Among patients with H5N1 virus infection, neutralizing antibodies are thought to persist for nearly 5 years,^5^ although few patients have been studied. Antibodies induced by natural infection with the 2009 pandemic H1N1 virus persist for at least 15 months.^6^ In our cohort of H7N9 patients,^7^ antibodies against H7N9 virus were detected in the majority of patients about one year after symptom onset, although the antibodies decayed over time. However, the duration of antibody responses in patients beyond 2 years has not been previously studied. In this report, we examined antibody responses against H7N9 virus among 14 patients from our prior cohort, 2 years after their symptom onset.^7^

## 2 METHODS

### 2.1 Study design and subjects

In our previous study,^7^ we enrolled 25 patients with laboratory‐confirmed H7N9 infection and studied changes in their antibody response to H7N9 virus over time (acute phase, 100, 200, and 300 days), and there were 22 patients followed up at 300 days after infection. In April 2019, using informed consent, 14 patients of these 22 participated in the subject follow‐up study, permitting serum collection approximately 2 years after symptom onset. A shorter questionnaire was used to collect information about their demographic characteristics, history of poultry exposure, their experience of influenza‐like illness (ILI), seasonal influenza vaccination, and medication use after the last follow‐up period. The Beijing Institute of Microbiology and Epidemiology's institutional review board approved the study.

### 2.2 Serological testing

The hemagglutination inhibition (HI) assay, the enzyme‐linked *lectin* assay to measure neuraminidase inhibition (NI) antibodies, and a microneutralization (MN) assay were used to measure antibodies as described in our previous study,^7^ We defined the HI titer as the reciprocal of the highest serum dilution that completely inhibited hemagglutination, the NI titer as the reciprocal of the highest serum dilution that exhibited 50% inhibition concentration, and the MN titer as the reciprocal of the highest serum dilution that yielded >50% neutralization. For final titers <10 of HI, NI, and MN antibodies, we assigned a value of 5 as seronegative, and a titer ≥40 was reported as 50% protective threshold.

### 2.3 Virus strains

A H7N9 virus (A/Jiangsu/Wuxi05/2013) and a genetic reassortant H6N9 virus (contains the hemagglutinin gene of H6N1 virus A/Taiwan/1/2013, the neuraminidase gene of H7N9 virus A/Anhui/1/2013, and other internal genes of A/Puerto Rico/8/1934 H1N1) used in our previous study^7^ were employed for the HI, MN, and NI assays.

### 2.4 Quality control

Although this study is the continuation of our previous work reported, and assays for antibodies detection were consistent in both study,^7^ the time of detection was not synchronized, which may lead to variation in results. Thus, considering the variation and the specificity of the assays to measure antibodies to H7N9 virus, five serum samples from these 14 patients at each time point of acute phase, 100, 200, and 300 days after infection and five serum samples from control subjects in our previous study were used as positive and negative controls when testing the serum samples from these 14 patients.

## 3 RESULTS

Among 22 patients who participated in the last follow‐up visit (about 1 year after infection),^7^ 14 consented to this new follow‐up testing, with a median follow‐up of 850 days (interquartile range 841‐865) after symptom onset. Participants ranged from 41 to 77 years of age (median 60.5 years), and 6 (42.9%) were female (Table 1). Two (14.3%) participants had a known exposure to poultry both within 14 days and one year before sampling. Two (14.3%) participants experienced ILI within 1 year before sampling; 12 (85.7%) reported taking medication during the last years because of hypertension, diabetes, heart disease, or other diseases.

**Table 1:** Epidemiological characteristics of influenza A(H7N9) virus survivors, China, 2019

Patient No.	Age (y) and gender	Poultry exposure		Influenza‐like illness		Influenza vaccination	Received medicine/Have an underlying Diseases	
		Preceding 14 d	Preceding 1 y	Preceding 1 mo	Preceding 1 y			
3	44/F	No	No	No	No	No	Yes/Microadenoma	
8	57/F	No	No	No	Yes	Yes	Yes/Hypertension, pneumonia	
9	56/F	No	No	No	No	No	No/No	
10	62/F	No	No	No	No	No	Yes/Urinary tract infection	
13	67/M	No	No	No	No	No	Yes/Hypertension	
15	41/F	No	No	Yes	No	No	Yes/Influenza‐like illness	
17	77/M	No	No	No	No	No	Yes/Hypertension	
18	60/M	No	No	No	No	No	Yes/Hypertension	
19	56/F	No	No	No	No	Yes	No/No	
21	70/M	Chicken	Chicken	No	No	No	Yes/Hypertension, diabetes	
22	61/M	No	No	No	No	No	Yes/Hypertension	
23	47/M	No	No	No	No	No	Yes/Hypertension	
24	73/M	No	No	No	No	No	Yes/Hypertension, heart disease	
25	66/M	Chickens, pigeons	Chickens, pigeons	No	No	No	Yes/Hypertension, stroke	

Note

Patient numbers match those in our previous study.^7^

The geometric mean titers (GMTs) and the proportion of survivors who had titers ≥40 for HI, NI, and MN antibodies approximately 2 years after infection were shown in Table 2. Thirteen (92.9%) patients had positive HI titers ranging from 10 to 40, and only 3 (21.4%) of 14 patients had a titer ≥40. The GMT of HI antibody was 20 (95% CI 15.7‐28.1) and below the titer of 40. The NI titer ranged from 10 to 80, and 10 (71.4%) of 14 patients had a titer ≥40. Similar to HI antibody, the GMT (34.44, 95% CI 25.7‐51.2) of NI antibody was also below titer of 40. Unlike the HI and NI antibody titers, all survivors had MN antibodies titers of ≥40 about 2 years after symptom onset, and yet GMTs of MN antibodies remained considerably above titer of 40.

**Table 2:** Proportion of influenza A(H7N9) virus patients with titers ≥40 and geometric mean titers, approximately 2 y after infection, China, 2019

	Antibody		
	HI	NI	MN
% (95% CI)^a^	21.4 (4.7‐50.8)	71.4 (41.9‐91.6)	100 (76.8‐100)
GMT (95% CI)	20 (15.7‐28.1)	34.44 (25.7‐51.2)	73.45 (54.7‐106.7)

Abbreviations: CI, confidence interval; GMT, geometric mean titers; HI, hemagglutination inhibition; MN, microneutralization; NI, neuraminidase inhibition.

a The proportion and 95% CI of patients with HI, NI, and MN titers ≥40.

**Figure 1:**
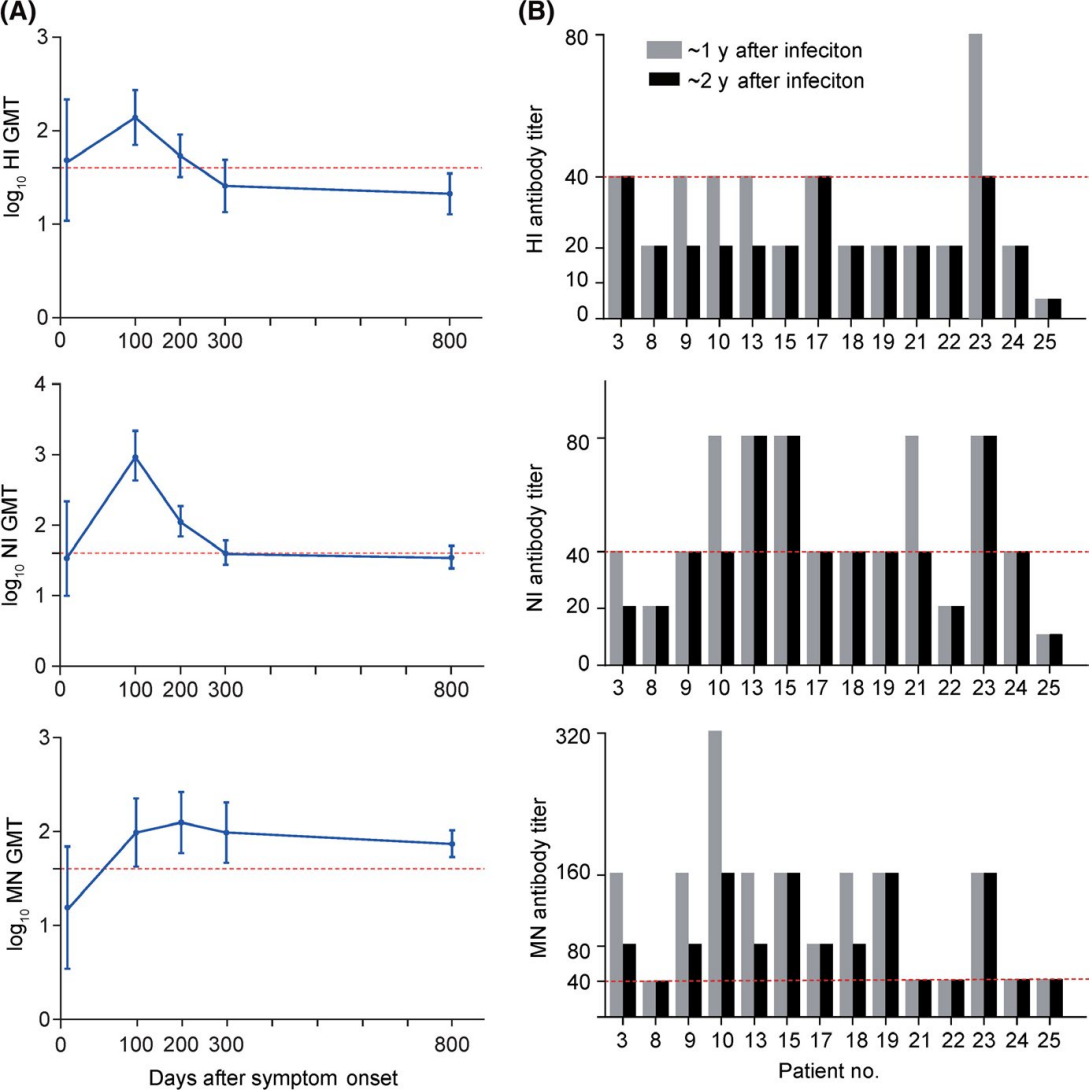
GMTs (A) and quantitation of antibody titer (B) against H7N9 virus among 14 surviving patients 2 y after infection, China, 2019. The GMTs of these 14 patients at acute phase, 100, 200, and 300 d after infection from our previous study 7 were used to show the dynamic changes over time, and the antibody titers at approximately 300 d after infection were used to compare with the antibody titers about two years after infection. Patient numbers match those in Table 1. The red dashed line indicates titer = 40 for HI, NI, and MN antibodies. Error bars indicate 95% CIs. GMT, geometric mean titer; HI, hemagglutination inhibition; MN, microneutralization; NI, neuraminidase inhibition

To show the time course of antibodies of these 14 patients after infection in the acute phase and four‐time follow‐ups, we used data from our previous study,^7^ and the GMTs of antibodies were plotted by the time points in Figure 1A. Overall, approximately 2 years after symptom onset, HI and NI GMTs substantially declined and were lower than the titer of 40 and the GMTs in the acute phase. In contrast, although the MN GMTs declined 2 years after infection, yet they remained considerably above the GMTs in the acute phase and the titer of 40. We further examined the changes in antibody titers approximately 2 years after symptom onset in comparison with the titers at 300 days after symptom onset. Most patients maintained antibody titers compared with their titers 300 days after infection, but HI assay titers were lower ranging from 5 to 40, while NI and MN antibodies were relatively high, ranging from 10 to 80 and 40 to 160, respectively. In particular, four patients (patients 9, 10, 13, and 23) experienced a decrease in HI antibody titer, three (patients 3, 10, and 21) experienced a decrease in NI antibody titer, and five (patients 3, 9, 10, 13, and 18) experienced a decrease in MN antibody titer approximately 2 years after symptom onset. However, none of these patients had antibody titers that were considered as seronegative.

## 4 DISCUSSION

In this study, we examined the levels of virus‐specific antibodies approximately 2 years after infection. Detectable levels of HI, NI, and MN antibodies were observed in all but one patient who has already had a HI antibody titer <10 at the last follow‐up visit (about 300 days after infection). While over 70% of patients maintained NI and MN titer ≥40 2 years after infection, this was observed in only 21.4% of patients for HI antibody. We also found that most patients (10 for HI antibody, 11 for NI antibody, and 9 for MN antibody) maintained antibody titers compared with their titers 300 days after infection. Although few patients had a decrease in one or more antibody titers, no patient had a decrease in antibody titer to become totally seronegative. These results suggest that natural infection of the H7N9 virus could induce the persistence of antibody response at least 2 years after infection.

An HI titer of 40 is associated with 50% protection of patients from seasonal influenza illness.^8^, ^9^, ^10^ Evidence for the contribution of NI antibody to the protection against seasonal influenza, independent of the effect of HA antibody, has been suggested by patterns of infection during the 1968 pandemic and more recently using multivariable regression analyses.^11^, ^12^, ^13^ Given the relative high NI titers observed here in comparison with HI titers, natural infection response to NA might have a longer duration of protection even when HA drifts, but NA does not. Based on the protective effect of MN observed for seasonal influenza, if MN antibody is indeed a better correlate for protection than HI antibody, we could anticipate that a proportion of H7N9 patients would remain protected against the H7N9 virus at about 2 years after the initial infection, but has not been proven so.

Data on immune responses following human infections with H7N9 virus remain somewhat limited. In particular, the levels and duration of antibody response after the infection are unknown. To our knowledge, measurement of antibody response to H7N9 virus in 14 patients of our previous cohort 2 years after infection is the longest longitudinal study of H7N9 patients ever reported. Previous longitudinal studies have reported antibody response of H7N9 patients, but only a few had complete dataset (2‐9 patients) and shorter follow‐up periods (102‐375 days).^14^, ^15^ In our previous cohort study,^7^ 22 patients who were laboratory‐confirmed H7N9 virus infection were prospectively evaluated the dynamic changes in their antibody response at the acute phase, 100, 200, and 300 days after symptom onset. In this study, 14 of these 22 patients were followed up. Although the sample size is small, this is the largest cohort of H7N9 patient to study long‐term antibody response of H7N9 patients, and all 14 patients had a complete dataset. In conclusion, this study provides evidence that infection with the H7N9 virus could induce long‐term antibody response at least 2 years after infection.

## CONFLICT OF INTEREST

The authors declare no conflict of interest.
